# Risk factors for rapid kidney function decline in diabetes patients

**DOI:** 10.1080/0886022X.2024.2398188

**Published:** 2024-09-11

**Authors:** Jixin Xing, Linxi Huang, Weifu Ren, Xiaobin Mei

**Affiliations:** a Department of Nephrology, Changhai Hospital, Naval Medical University (Second Military Medical University), Shanghai, China; b Department of Nephrology, PLA Navy No. 905 Hospital, Naval Medical University (Second Military Medical University), Shanghai, China

**Keywords:** Diabetic nephropathy, rapid decline in kidney function, genetic factors, clinical risk factors

## Abstract

Diabetic nephropathy, as a severe microvascular complication of diabetes, manifests in four clinical types: classic, albuminuria regression, a rapid decline in kidney function (RDKF), and non-proteinuric or non-albuminuric DKD. Rapidly progressive diabetic nephropathy advances to end-stage renal disease more swiftly than the typical form, posing significant risks. However, a comprehensive understanding of rapidly progressive diabetic nephropathy is currently lacking. This article reviewed latest developments in genetic and clinical risk factors associated with rapidly progressive diabetic nephropathy, aiming to broad perspectives concerning the diagnosis and interventions of this condition.

## Introduction

Diabetes mellitus (DM) is one of the largest global public health problems, and the prevalence continues to increase at an alarming rate [1,2]. The International Diabetes Federation (IDF) has estimated that in 2017 there were 451 million people living with DM worldwide, which figure was expected to reach 693 million by 2045 with the absence of effective control [3]. DM causes damages in multiple organs including the heart, eyes, nerves, and kidneys, resulting in various complications and death [4]. In the case of kidneys, the metabolic and hemodynamic disturbances caused by DM lead to glomerular hyperfiltration, damage the glomeruli (especially the pedunculated cells), and subsequent interstitial fibrosis and tubular atrophy [5]. As disease progresses, persistent albuminuria and a decrease in the estimated glomerular filtration rate (eGFR) appear, which is diagnosed with diabetic kidney disease (DKD) [6]. DKD occurs in nearly 30–40% of DM patients, and has become the leading cause of chronic kidney disease (CKD) and end-stage renal disease (ESRD) globally, which costs millions in medical expenditures and bears heavy burden [7,8].

**Figure 1.**
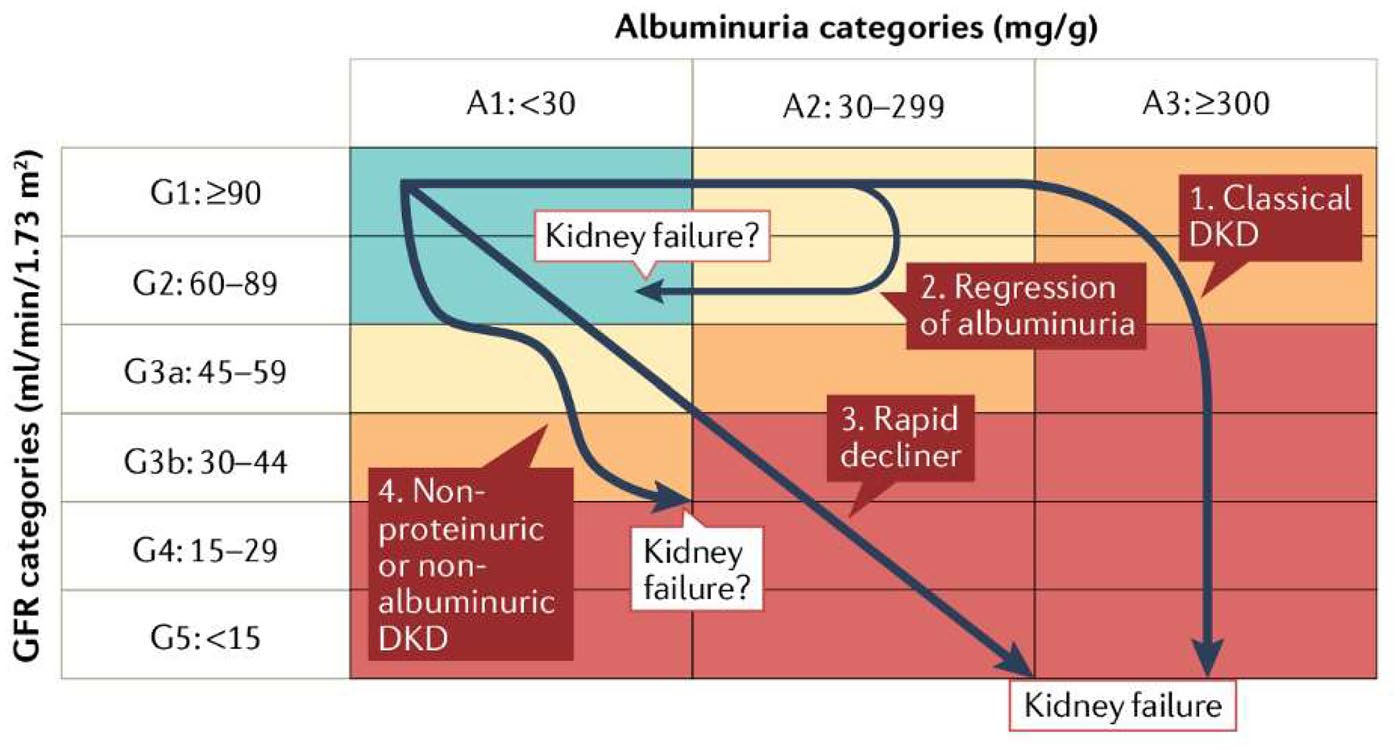
Four types of kidney function trajectories in DKD [9].

Recent studies reported four DKD phenotypes characterized by different trajectories of kidney functions in patients with diabetes, i.e., classic, albuminuria regression, a rapid decline in kidney function (RDKF), and non-proteinuric or non-albuminuric DKD [9] (Figure 1). The classical type is characterized by a supraphysiological elevation in GFR in early history and subsequent irreversible nephron damage, which caused microalbuminuria (urinary albumin-to-creatine ratio, UACR, 30–300 mg/g), maroalbuminuria (UACR > 300 mg/g) and finally ESRD [5]. The albuminuria regression happened in some diabetic patients. The treatment of antihypertensive drugs, particularly angiotensin-I converting enzyme (ACE)-inhibitors, and sodium-glucose cotransporter 2 inhibitors probably induced the remission [10–12]. The DKD type without proteinuria or albuminuria characterized a group of diabetic patients with normoalbuminuria whilst progressive GFR decline [13]. The possible explanation is the underlying pathologic features like predominant lesions in (macro)vessels and tubulo-interstitium instead of glomerulus [14]. The rapid decliners often develop kidney failure over a short period of time regardless of the presence of albuminuria or proteinuria [9].

Studies have revealed that the annual eGFR slope is associated with high risk of future renal failure, cardiovascular disease, and all-cause mortality [15–17]. In that case, RDKF in DKD evoked wide attention with probable poor prognosis. According to KDIGO guidelines, the annual rate of eGFR decline as ≥5 mL/min/1.73 m^2^/year is termed with ‘rapid decline’ [18]. Nevertheless, investigators also defined ‘rapid GFR decline’ as −3 mL/min/1.73 m^2^/year, −3.5 mL/min/1.73 m^2^/year, −3.3%/year or −10%/year in different researches [19–22]. Thus, no consensus has been reached on the prevalence of rapid GFR decline in diabetic patients. Data from Northern Europe involving 4606 patients found that 32.8% of patients with T2DM and 14% of patients with T1DM experienced a rapid decline in eGFR over a median follow-up of 5.48 years [23]. Another study in Japan of 377 biopsy-proven diabetic nephropathy reported 61% of patients experienced rapid eGFR decline during 6.9 years’ follow-up [24]. An observational study in Japan reported 14% of 1955 patients with type 2 diabetes had a rapid eGFR decline over 3 years [25]. The great variations may also stem from the diversities in clinical interventions and monitors in blood pressure, glucose control, urine protein, and renal functions. Individual heterogeneity further contributed to the inconsistency in present studies.

Nevertheless, researchers also revealed some clues in rapid eGFR decliners of diabetic patients. Macroalbuminuria often predicts rapid eGFR decline while the progression of albuminuria is sometimes bidirectional and albuminuria regression can occur [9,26]. Meanwhile, serum markers like adipocyte fatty acid-binding protein, lysophosphatidylcholine, tumor necrosis factor receptors, and KIM-1 were reported to be promising biomarkers in the refinement of rapid decliners in DKD patients [26–28]. Renal pathological features as glomerulosclerosis, interstitial fibrosis, tubular atrophy, nodular lesions, mesangiolysis, and arteriolar hyalinosis were also reported to be significantly associated with rapid eGFR decline in diabetes patients [20,24,29,30].

**Figure 2.**
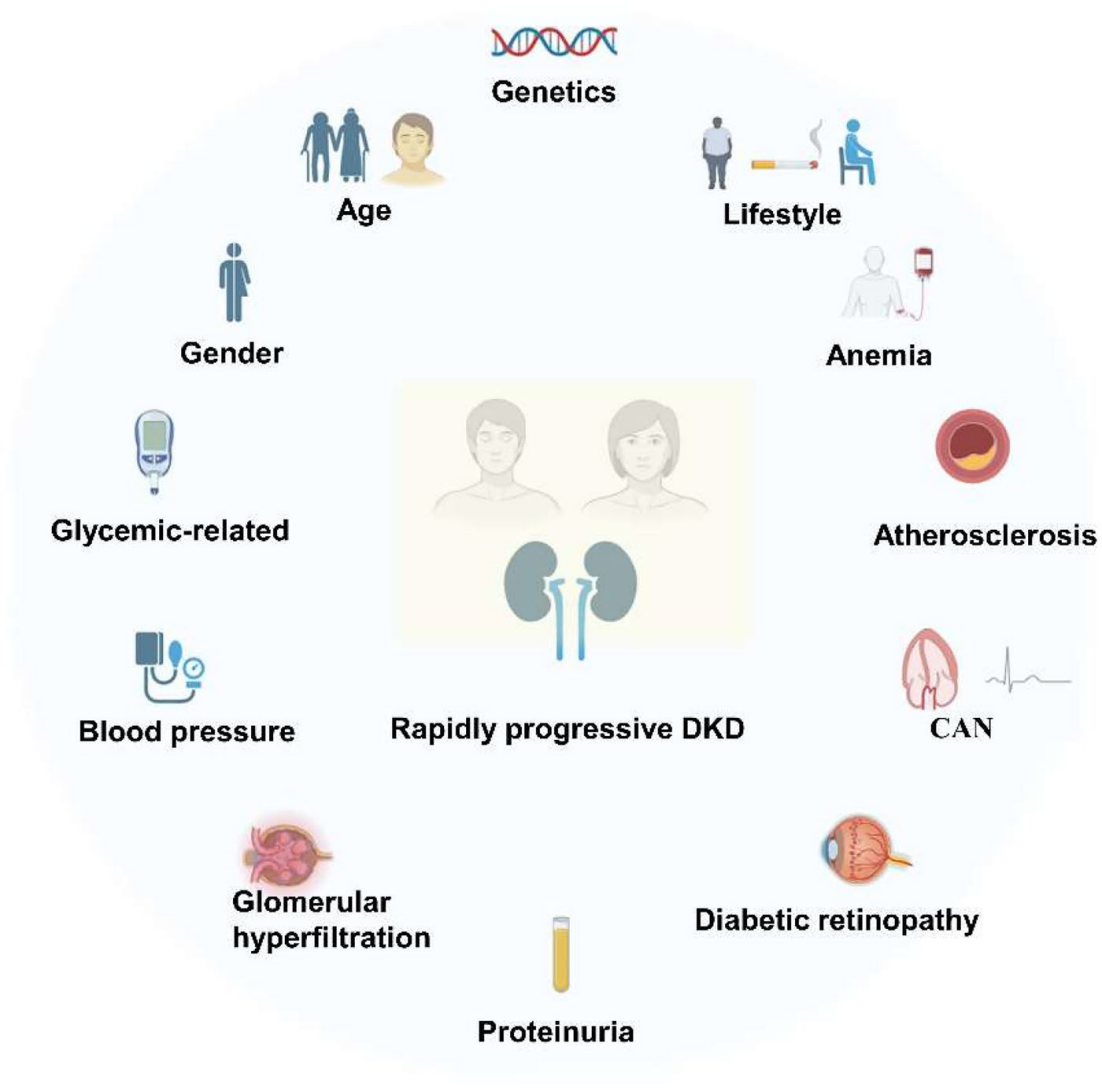
Risk factors for rapidly progressive kidney disease in diabetes patients [31].

Given the fact that rapid renal function decline is associated with a slope into ESRD, and a marked increase in cardiovascular events, heart failure risk, and all-cause mortality, the urgent requirements emerged with intensified researches on rapidly progressive DKD to further understand the pathogenesis, manage risk factors and clarify effective interventions. In this article, we will highlight recent advances in genetic and clinical findings in relation to rapidly progressive DKD (Figure 2). With comprehensive search and reading of related studies, we summarized the association strength of potential risk factors basically based on the quality of supportive studies (Table 1). Table 2 also presented the details of enrolled studies, hoping to better clarify solid evidences and inspire future researches.

**Table 1.** The association strength of discussed risk factors.

The strength of association	Risk factors
Very likely	Genetics, poor glycemic control, intensive blood pressure control, hypertension, lifestyle
Probable	Older age, rapid glycemic decline, CAN, anemia
Possible	Early-onset type 2 diabetes, atherosclerosis, diabetic retinopathy, proteinuria
Controversial	Gender

**Table 2.** The characteristics of supportive studies.

	Author (year)	Research type	Number	Age (years)	Male, *n* (%)	Follow-up (years)	Group *n* (%)	Results
Genetics	Yiting Wang et al. 2019 [32]	Retrospective cohort study	128	51.5 ± 10.68	97 (76)	2.0 (1.4–3.2)	Rapid decliners (< −8.1 mL/min/1.73 m^2^/year). 64 (50)	Multivariable logistic regression indicated that a family history of diabetes (OR = 3.973, 95% CI 1.220–12.931, *p* = 0.022) was independently associated with a rapid decline in eGFR.
	Scott G. Frodsham et al. 2019 [33]	Retrospective cohort study	15,612	47 (38–54)	8316 (53.3)	5.7 (3.1–10.6)	Rapid decliners (< −5 mL/min/1.73 m^2^/year). 2127 (13.6)	The OR of rapid renal decline among first-degree relatives of rapid decliners to control diabetes subjects with slow renal decline was 1.92 (*p* = 0.007 [95% CI 1.19–3.09]).
	Guozhi Jiang et al. 2019 [34]	Prospective cohort study	6330	54.3 ± 12.4	3019 (47.7)	13.0 (9.5–16.8)	Accelerated eGFR decline (the median rates of decline 14.3% [11.3–17.6%] per year) 197 (3.1)	Of 68 candidate genetic loci evaluated, the inclusion of five loci improved the prediction of eGFR trajectories (net reclassification improvement 0.232; 95% CI: 0.057–0.406)
	Resham Lal Gurung et al. 2021 [35]	Prospective cohort study	1337	SMART2D 57.12 ± 11.50 DN 54.31 ± 12.43	SMART2D 391 (52.2) DN 362 (61.6)	SMART2D 5.62 ± 1.99 DN 7.56 ± 3.94	Rapid decliners (< −3 mL/min/1.73 m^2^/year) 344 (26)	The rs4806985 variant was associated with higher odds of RDKF (meta OR = 1.23, *p* = 0.030 adjusted for age and sex). Mendelian randomization analysis provided evidence for a potential causal effect of plasma LRG1 on kidney function decline in T2D (*p* < 0.05).
	Yu Wu MD et al. 2023 [36]	Prospective cohort study	1241	61.6 ± 9.7	791 (63.7)	1	RDKF (< −3 mL/min/1.73 m2/year) 441 (35.5)	The rs285 C allele was associated with higher odds of RDKF (OR = 1.31, 95% CI 1.04–1.66; *p* = .025) after adjustment for multiple variables.
	Resham Lal Gurung et al. 2022 [37]	Prospective cohort study	1146	SMART2D 56.5 ± 11.6 DN 54.5 ± 12.3	SMART2D 349 (52.2) DN 299 (62.6)	6.55 (4.81–7.62); 6.86 (4.37–10.87)	RDKF (< −5 mL/min/1.73 m2/year) 160 (14.0)	Higher wGRS was associated with increased odds for RDKF (meta-adjusted odds ratio = 1.12, [95% CI, 1.01–1.24], *p* = 0.030, Phet = 0.606).
Older age	Giacomo Zoppini et al. 2012 [38]	Prospective cohort study	1682	65.1 ± 9	1038 (61.7)	10	Rapid eGFR decline (>4.0% per year) 263 (15.6)	Rapid decliners were significantly older (67 ± 9 *vs.* 64 ± 9, *p* < 0.001). The annual eGFR decline was independently (*p* < 0.05–0.001) related to age (standardized coefficient: −0.07)
	Yui Yoshida et al. 2020 [25]	Cross-sectional, prospective cohort study	1955	NR	NR	3	Early decliners (the group whose eGFR decreased most) 274 (14)	Age (by 10 years) of early decliners was calculated by multiple regression analysis (Regression coefficient −0.44, [95% CI, −0.65 to −0.23]). Age (by 10 years) of the rapidly lowering group by trajectory analysis (OR1.38, [95% CI, 1.19–1.60])
Early-onset type2 diabetes	Jian-Jun Liu et al. 2018 [39]	Prospective cohort study	1189	56.0 ± 11.2	635 (53.4)	3.9 (3.2–4.7)	Progressive CKD (>5 mL/min/1.73 m^2^ per year) Early-onset 38 (24.2) Later-onset 161 (15.6)	Logistic regression suggested that participants with early-onset T2DM had 2.63-fold [95%CI 1.46–4.75] higher risk of progressive CKD after accounting for multiple traditional risk factors.
Gender	Fan Zhang et al. 2024 [40]	Retrospective cohort study	1549	51.00 (41.00–61.00)	1006 (64.9)	5	Male 1006 (64.9) Female 543 (35.1)	Multiple linear regression analysis revealed that female gender had a significant impact on ΔeGFR% in T2DM patients (standardized β-0.219,[95%CI −8.906, −5.786], *p* < 0.001)
	Da Hea Seo et al. 2019 [41]	Prospective cohort study	967	53.7 ± 9.4	555 (57.4)	6	Rapid decline (>3.3% per year) 158 (16.3)	The decline was more rapid in female (−2.1 ± 2.5%/year) than male (–0.4 ± 2.4%/year; *p* < 0.001) Female sex was a predictor of rapid renal function decline (OR, 3.46; 95% CI, 2.19–5.47; *p* < 0.0001).
	A. de Hauteclocque et al. 2014 [19]	Prospective cohort study	1146	65 ± 11	660 (57.6)	5.7 (0.1–10.2)	Rapid decliners (< −3.5 mL/min/1.73 m^2^/year) 361 (31.7)	Multivariable logistic regression indicated that male sex was an independent risk factor of steep eGFR decline [adjusted OR = 1.33 (1.02–1.76), *p* = 0.04].
Poor glycemic control	Hetal S. Shah et al. 2024 [42]	Randomized controlled trial	PERL 531 ACCORD 2378	PERL 51 ACCORD 64.4	PERL [1] 106 (67.1); [2] 116 (65.2); [3] 130 (66.7) ACCORD [1] 342 (69.8); [2] 686 (70.6); [3] 633 (69.1)	NR	PERL: HbA1c [1] ≤ 7.5% 158 (29.8); [2] = 7.5–8.5% 178 (33.5); [3] ≥ 8.5% 195 (36.7) ACCORD: HbA1c [1] ≤ 7.5% 490 (20.6); [2] = 7.5–8.5% 972 (40.9); [3] ≥ 8.5% 916 (38.5)	A higher baseline HbA1c was associated with a more negative eGFR slope in both PERL and ACCORD (−0.87 and −0.27 mL/min/1.73 m^2^/year per Hba1c unit increment, *p* < 0.0001 and *p* = 0.0002, respectively).
Rapid glycemic decline	Lingwang An et al. 2021 [43]	Retrospective cohort study	2599	59.7 (53.2–65.3)	1514 (58.3)	0. 7(0.5–0.92)	Fast decline (< −10 mL/min/1.73 m^2^/year) 821 (31.6)	An eGFR FD was frequently found in patients who had an HbA1c reduction of ≥3.00% and a baseline HbA1c ≥ 8.0%.
Hypertension	Makoto Fujii et al. 2023 [22]	Prospective cohort study	3,673,829	Diabetes 60.02 ± 9.03 No diabetes 57.34 ± 9.64	2,139,173 (58.2)	1	Rapid decline (−10%/year decline) 542,758 (14.8)	Participants with an SBP ≥ 120 mmHg and <130 mmHg had a higher risk of rapid decline than those with an SBP < 120 (OR = 1.10, 95% CI 1.07–1.13; *p* < 0.001). The ORs also increased with poorer BP control.
	Giacomo Zoppini et al. 2012 [38]	Prospective cohort study	1682	65.1 ± 9	1038 (61.7)	10	Rapid eGFR decline (eGFR decline > 4.0% per year) 263 (15.6)	Participants with high systolic blood pressure had a higher risk of rapid decline (142 ± 19 *vs.* 137 ± 18, *p* < 0.001). The annual eGFR decline was independently (*p* < 0.05–0.001) related (standardized coefficient: −0.10).
	Yi-Jing Sheen et al. 2014 [44]	Prospective cohort study	215	61 ± 11	117 (54.4)	1	Rapid decliners (< −5 mL/min/1.73 m^2^/year) 44 (20.5)	Systolic blood pressure (SBP) was found to be significant independent factors for a rapid decline of eGFR in model (OR = 1.023, 95% CI:1.001–1.046, *p* = 0.042).
Intensive blood pressure control	S. Beddhu et al. 2019 [45]	Randomized controlled trial	6662	66.3 ± 9.0	4418 (66.3)	3.3 (0–4.8)	Intensive group: SBP goal 120 mmHg 3326 (50) Standard group: SBP goal 140 mmHg 3336 (50)	The difference between groups was −3.32 mL/min/1.73 m^2^ (95%CI, −3.90 to −2.74 mL/min/1.73 m^2^) at 6 months, was − 4.50 mL/min/1.73 m^2^ (CI, −5.16 to −3.85 mL/min/1.73 m^2^) at 18 months.
Glomerular hyperfiltration	Wei-Lun Wen et al. 2024 [46]	Retrospective cohort study	7563	Hyperfiltration 50.7 ± 10.8 Normofiltration 56.1 ± 11.5	3870 (51.1)	4.65 ± 3.86	Renal hyperfiltration (eGFR exceeding the age- and gender-specific 95th percentile values) 543 (7.2)	The hyperfiltration group exhibited a higher rate of eGFR decline (−2.0 ± 0.9 *vs.* −1.1 ± 0.9 mL/min/1.73 m^2^/year; *p* < 0.001). Cox regression analyses identified that initial hyperfiltration was a significant determinant of rapid DKD progression, with a hazard ratio of 1.66 (95%CI: 1.41–1.95; *p* < 0.001).
	Petter Bjornstad et al. 2015 [21]	Prospective cohort study	519	RD 34 ± 9 No RD 37 ± 9	228 (43.9)	6	Renal hyperfiltration (eGFR ≥ 120mL/min/1.73 m^2^) 147 (28.3)	Renal hyperfiltration predicted greater odds of rapid GFR decline (>3 mL/min/1.73 m^2^) [OR: 5.00, 95%CI: 3.03–8.25, *p* < 0.0001].
	Tatsumi Moriya et al. 2016 [47]	Prospective cohort study	1407	59 ± 7	740 (52.6)	8	G1 (120 ≤ eGFR) 157 (11.2) G2 (90 ≤ eGFR < 120) 355 (25.2) G3 (60 ≤ eGFR < 90) 735 (52.2) G4 (eGFR < 60) 160 (11.4)	The risk of annual eGFR decline rate ≥ 3ml/min/1.73 m^2^ (rapid decliners) increased as the baseline eGFR increased. The G1 group had a significant risk for the rapid decliners.
	Serena Low et al. 2018 [48]	Prospective cohort study	1014	54.1 ± 11.1	617 (60.9)	6.0 ± 2.2	Glomerular hyperfiltration (eGFR ≥ 120 mL/min/1.73 m^2^) 53 (5.2)	Baseline hyperfiltration was significantly associated with greater odds of RD (≥3 mL/min/1.73 m^2^) (odds ratio: 2.57; 95%CI: 1.21–5.46; *p* = 0.014).
	Piero Ruggenenti et al. 2012 [49]	Retrospective cohort study	600	61.3 ± 7.8	NR	4.0 (1.7–8.1)	Glomerular hyperfiltration (eGFR ≥ 120 mL/min/1.73 m^2^) 90 (15)	At multivariable analysis, higher GFR at inclusion (coefficient [SE]: −0.006 [0.0007]; *p* < 0.0001) predicted a faster GFR decline throughout the whole study period.
Proteinuria	Andrzej S. Krolewski et al. 2017 [50]	Cross-sectional and prospective cohort study	364	Very fast 32 (27–38), Fast 34 (28–40), Moderate 33 (25–40), Slow 33 (29–42)	Very fast 36 (40.5) Fast 46 (58.2) Moderate 69 (56.1) Slow 35 (48.0)	6.6 (4.2–9.9)	Very fast (< −15 mL/min/1.73 m^2^/year) 89 (24.5) Fast (< −10 mL/min/1.73 m^2^/year) 79 (21.7) Moderate (< −5 mL/min/1.73 m^2^/year) 123 (33.8) Slow (> −5 mL/min/1.73 m^2^/year) 73 (20.0)	Studies from Joslin clinic reported that in patients with T1DM, the proportion of rapid decliners was 3, 11, and 35%, respectively when confounded with non-albuminuria, microalbuminuria or macroalbuminuria. The data was 7, 15, and 51% in patients with T2DM.
	Giacomo Zoppini et al. 2012 [38]	Prospective cohort study	1682	65.1 ± 9	1038 (61.7)	10	Rapid eGFR decline (eGFR decline > 4.0% per year) 263 (15.6)	Participants with proteinuria had a higher risk of rapid decline (microalbuminuria [%] 23.4 *vs.* 15.9, *p* < 0.001; macroalbuminuria [%] 9.6 *vs.* 2.9, *p* < 0.001) The annual eGFR decline was independently (*p* < 0.001) related to albuminuria (standardized coefficient: –0.16).
Diabetic retinopathy	Roberto Trevisan et al. 2002 [51]	Prospective cohort study	65	DR 57 ± 8 No DR 55 ± 8	DR 30 (78.9) No DR 21 (77.8)	DR 5.96 (2–10) No DR 6.13 (2–10)	Patients with retinopathy 38 (58.5) Patients without retinopathy 27 (41.5)	The rate of decline of GFR was higher in type 2 diabetic patients with retinopathy (−6.5 ± 4.4 ml/year) than in those without (−1.8 ± 4.8 ml/year; *p* < 0.0001).
	Hayne Cho Park et al. 2019 [52]	Retrospective cohort study	1592	57.9 ± 11.2	751 (47.2)	5.6 ± 2.1	No DR 1006 (63.2) Non-proliferative DR 384 (24.1) Proliferative DR 202 (12.7)	Patients with NPDR (*n* = 102) and PDR (*n* = 51) showed faster eGFR decline rate (−3.2 ± 6.44 and −4.16 ± 5.43 mL/min/1.73 m^2^ per year) compared to those without DR (*n* = 364, −0.83 ± 3.48 mL/min/1.73 m^2^ per year, *p* < 0.001).
Cardiovascular autonomic neuropathy	Steven Orlov et al. 2015 [53]	Retrospective cohort study	370	CAN 34 ± 6 No CAN 30 ± 7	NR	2	CAN 47 (12.7) No CAN 323 (87.3)	Early GFR loss (< −3.3%/year) occurred in 15 (32%) of the 47 patients with baseline autonomic neuropathy and in 32 (10%) of the 323 without baseline autonomic neuropathy (*p* < 0.001). Baseline autonomic neuropathy was strongly associated with odds of early GFR loss (adjusted OR = 4.09; 95%CI 1.65–10.12; *p* = 0.002).
	Abd A Tahrani et al. 2014 [54]	Prospective cohort study	204	No CAN 54.2 ± 12.1 CAN 59.5 ± 11.4	No CAN 71 (60.2) CAN 52 (60.5)	2.5	No CAN 118 (57.8) CAN 86 (42.2)	eGFR declined to a greater extent in patients with CAN than in those without CAN (−9.0 ± 17.8 *vs.* −3.3 ± 10.3%, *p* = 0.009). After adjustment for baseline eGFR and baseline differences, CAN remained an independent predictor of eGFR decline (*β* = −3.5, *p* = 0.03)
	Yaling Tang et al. 2024 [55]	Prospective cohort study	PERL 469 ACCORD 7793	PERL CAN 51.0 ± 10.8 No CAN 51.0 ± 11.3 ACCORD CAN 62.6 ± 6.4, no CAN 62.3 ± 6.7	PERLCAN 193 (65.9), No CAN 116 (65.9) ACCORD CAN 3122 (61.1), no CAN 1522 (58.9)	PERL 3.2 (3.1–3.3) ACCORD 4.9 (4.0–5.7)	PERL CAN 293 (62.5) No CAN 176 (37.5) ACCORD CAN 5183 (66.5) No CAN 2610 (33.5)	Participants with CAN experienced more rapid GFR decline, by an excess 1.15 mL/min/1.73 m^2^/year (95% CI −1.93 to −0.37; *p* = 4.0 × 10^−13^) in PERL and 0.34 mL/min/1.73 m^2^/year (95%CI −0.49 to −0.19; *p* = 6.3 × 10^−6^) in ACCORD. This translated to 2.11 (95%CI 1.23–3.63; *p* = 6.9 × 10^−3^) and 1.39 (95% CI 1.20–1.61; *p* = 1.1 × 10^−5^) OR of rapid kidney function decline in PERL and ACCORD.
Atherosclerosis	Miriam Goepfert et al. 2023 [56]	Prospective cohort study	2904	54 (41–66)	1399 (48.2)	14	cIMT > 0.71 mm 1429 (49.2) cIMT < 0.71 mm 1475 (50.8)	Subjects with high cIMT and the presence of plaques at baseline showed a greater decrease in eGFR (cIMT: FAS-eGFR: *p* < 0.001, CKD-EPI-eGFR: *p* < 0.001; plaques: FAS-eGFR: *p* < 0.001, CKD-EPI-eGFR: n.s.)
	Da Hea Seo et al. 2019 [41]	Prospective cohort study	967	53.7 ± 9.4	555 (57.4)	6	Rapid decline (>3.3% per year) 158 (16.3)	In multivariable logistic regression analysis, presence of carotid plaque was an independent predictor of rapid renal function decline (OR: 2.33; 95%CI 1.48–3.68; *p* < 0.0001).
Anemia	Makoto Fujii et al. 2023 [22]	Prospective cohort study	3,673,829	Diabetes 60.02 ± 9.03 No diabetes 57.34 ± 9.64	2,139,173 (58.2)	1	Rapid decline (−10%/year decline) 542,758 (14.8)	Participants with Hb levels ≥11 and <13 mg/dL had a higher risk of rapid decline than those with Hb levels ≥13 mg/dL (OR = 1.41, 95% CI: 1.33, 1.50; *p* < 0.001). The ORs also increased with lower Hb levels.
	Lijie Xie et al. 2023 [57]	Retrospective cohort study	526	RD 65.5 (59–76) Slow decline 68 (59–77)	RD 151 (56.3) Slow decline 148 (57.4)	2.75	Rapid eGFR decline (< −5 mL/min/1.73 m^2^/year) 268 (51.0)	Logistic regression analysis showed that anemia was an independent risk factor for rapid eGFR decline. [OR = 1.66,95%CI 1.02–2.69, *p* = 0.041]
	Daishi Hirano et al. 2023 [58]	Retrospective cohort study	242	59 (53–64)	182 (75.2)	6.7 (5.0–7.4)	Rapid decliners (≥3.3%/year) 34 (14.0)	On multivariate analysis, lower baseline hemoglobin level was a risk factor of rapid decliners (odds ratio 0.69, 95% confidence interval 0.47–0.99; *p* = 0.045)
Lifestyle	Rieko Okada et al. 2021 [59]	Prospective cohort study	451,534	51.7 (20–79)	277,494 (61.5)	2	The number of healthy LFs 0: 16,138 (3.6) 1: 89,570 (19.8) 2: 181,093 (40.1) 3: 164,733 (36.5)	Subjects with a healthy lifestyle showed a 19% reduced risk of rapid eGFR decline (eGFR decline ≥ 20%) over 2 years. ORs for an eGFR decline ≥ 20%, 0.93 (95% CI 0.82–1.05),0.90 (0.79–1.02) and 0.81 (0.70–0.93) for those with one, two and three healthy LFs compared with those with none of the three healthy LFs.
	Yaya Li et al. 2023 [60]	Prospective cohort study	573,860	NR	443,607 (77.30)	2	Rapid decline eGFR (≥30% during the follow-up) 7683 (1.3)	In the baseline eGFR >30, skipping breakfast and regular smoking were associated with a rapid decline in both age groups.

## Genetic factors

Family history of diabetes in first-degree relatives is independently associated with rapid decline in eGFR either in American or in Chinese populations [32,33]. Further genome-wide association studies and whole-exome sequencing analysis identified genetic factors contributing to the severity of kidney phenotype in DKD patients [61–63]. One study on Chinese patients with type 2 diabetes identified 5 loci (rs11803049, rs911119, rs1933182, rs11123170, and rs889472) which were likely to predict an accelerated decline in kidney function [34]. Genetic variants at COL4A3, CLDN8, and ARHGAP24 were potentially pathogenic to the more severe kidney phenotype in DKD [62]. These studies highlighted the influence of shared familiar factors, particularly genetic factors on rapid renal decline I diabetes. Early diagnosis and interventions are required in clinical practices for these patients.

The aberrant transcriptive profiles in DKD kidneys were observed. Leucine-rich α-2-glycoprotein 1 (LRG1), an upregulated proangiogenic gene in DKD kidney, was found associated with worse renal outcome in patients with type 2 diabetes [64]. LRG1 localizes predominantly to glomerular endothelial cells, and both plasma and urine level can predict ESRD risk independent of other clinical factors in diabetic patients [65,66]. Genome-wide association study of plasma LRG1 in Singapore and Chinese cohorts further identified rs4806985 variant near LRG1 locus robustly associated with plasma LRG1 levels, which genetically influenced plasma level increases the risk of rapid decline in kidney function among patients with type 2 diabetes [35].

Other genetic polymorphisms also predict susceptibility to early renal function decline in diabetic patients. Lipoprotein lipase (LPL)-related single nucleotide polymorphism (SNP) rs285 C was identified to be significantly associated with higher risk of rapid decline in kidney function in type 2 diabetic patients [36]. Weighted genetic risk score (wGRS) analysis identified another 10 SNPs which was significantly associated with plasma uric acid level in Chinese T2D populations and further contributed to renal function decline. Among the 10 SNPs, rs2390793 (LRP2) was modestly associated with rapid decline in kidney function independent of other confounders [37].

Currently, the utilization of genetic tests and scores is limited in clinical practices. Nevertheless, the efforts in discriminating DKD patients with higher risk of RDKF characterized by specific genetic features helped a lot both in broadening our understanding of the pathological mechanisms and generating promising interventions in the future. On the other hand, environmental factors are easier to manage, which also contribute to disease progression. In that case, we still pay attention to demographic, glycemic, hemodynamic risk factors and so on to comprehensively capture the characteristics of RDKF type of DKD for benefits of patients.

## Demographic factors

### Older age

Studies have revealed that age was independently associated with renal impairment in T2D patients [67,68]. Yet, no available data supported the relationship of age and RDKF in diabetic patients. Moreover, the stratification of patients in advanced age (i.e., over 60 or 65 years old) is lack in present studies. A prospective, observational cohort study of 1682 T2DM patients found that rapid decliners were significantly older than non-decliners (67 ± 9 *vs.* 64 ± 9) [38]. Another cohort study in Japan also claimed older age was significantly associated with early decliners in DKD patients [25]. Older patients with diabetes are worthy of further focus for the high risk of RDKF and developing ESRD.

### Early-onset type 2 diabetes

T2DM in younger people (aged <40 years old), referred to as young-onset T2DM, has a more rapid deterioration of β-cell function, worse metabolic control, higher risk of complications, and premature mortality [69,70]. Large cross-sectional studies and observational cohorts also found that young-onset T2DM are complicated with higher prevalence of elevated ACR, chronic kidney disease, neuropathy, cardiovascular diseases, and even mortality [71–73]. Factors as insulin resistance, smoking, and genetics could be explanatory to these complications of early-onset diabetes [74,75].

Early-onset T2DM can lead to rapidly progressive DKD. A prospective cohort study followed 1189 T2DM patients for a median 3.9 years to profile the trajectory of renal function in early-onset T2DM patients. The authors defined the eGFR decline ≥5 mL/min/1.73 m^2^ per year as progressive CKD and found a significantly higher prevalence of progressive DKD in early-onset populations compared with later-onset counterparts (24.2 *vs.* 15.6%, *p* = 0.007). Logistic regression analysis predicted a 2.63-fold risk of rapid progressive DKD in early-onset T2DM patients. Furthermore, the study also found that renal function decline was more pronounced in diabetes duration <10 years [39]. These results suggest the rapid renal function decline associated with early-onset T2DM could not be solely attributed to longer diabetes duration and the potential distinct pathologic factors need to be further elucidated. In clinical practices, early and intensive monitor of renal function is warranted for early-onset T2DM populations.

### Gender

Men and women show sex-specific features in diabetes mellitus from prevalence, diagnosis age, risk factors, hormone levels, and complications [76]. Nevertheless, present studies did not generate consensus consulting the gender contribution to RDKF in DKD patients. In 2013, investigators conducted a meta-analysis, enrolled 46 cohorts from Europe, North and South America, Asia, and Australasia, and found no evidence of a sex difference in association with eGFR and ESRD risk [77]. But there is a different view that female could be a risk factor for RDKF. Female reproductive system profoundly effects a series of processes including energy homeostasis and metabolism [78]. A prospective cohort study of diabetic patients revealed that women, compared to men, had an increased prevalence of advanced DKD, i.e., eGFR <30 mL/min/1.73 m^2^ [79]. Another retrospective cohort study of 1549 T2DM patients concluded that women had a greater risk of developing DN and a faster decline in renal function compared to men [40]. A multicenter prospective cohort study also found that female sex is a significant predictor of rapid renal function decline [41]. Particularly, some studies found that female sex contributed to GFR deterioration in type 2 diabetes with normoalbuminuria [80,81].

Simultaneously, other studies reported male sex was associated with RDKF. Analysis from an inception cohort, SURDIAGENE, revealed that men were more likely than women to develop end-stage renal disease and male sex was an independent risk factor of steep estimated glomerular filtration rate decline [19]. Advances in basic research further revealed the potential underlying clues. A study published in 2024 found that male proximal tubular epithelial cells (PTECs) displayed increased mitochondrial respiration, oxidative stress, apoptosis, and greater injury when exposed to high glucose compared with PTECs from healthy females. Male human PTECs showed increased glucose and glutamine fluxes to the TCA cycle whereas female human PTECs showed increased pyruvate content, which phenotype was enhanced by dihydrotestosterone. Metabolomics analysis from adolescent cohort and adult cohort both showed increased TCA cycle metabolites in males and higher pyruvate concentrations in females. Moreover, serum pyruvate concentrations positively correlated with eGFR while plasma TCA cycle metabolites correlated with all-cause mortality. These findings suggest that metabolism differences contributed to sex differences in human DKD [82].

However, the contradictory conclusions from different studies may be associated with the diversities in risk factors of different genders of diabetic patients. Further studies need to stratify different conditions, such as smoking history, body mass index, menopause, history of hypertension, renal function, proteinuria, etc. to generate more accurate conclusions and unravel the precise mechanisms.

## Glycemic factors

### Poor glycemic control

The degree and duration of hyperglycemia were associated with the progression of CKD [83]. The risk of a rapid decline of glomerular function abruptly increases when glycated hemoglobin is steadily higher than 7.5% and postprandial blood glucose is over 200 mg/dL [84]. A study analyzed data from ACCORD and the Preventing Early Renal Loss (PERL) trial, and found that after the onset of DKD, poor glycemic control is associated with a more rapid rate of GFR decline, either in type 1 or type 2 diabetes [42]. As for the strategy to glycemic control, sodium-glucose cotransporter 2 (SGLT2) inhibitors show convinced kidney-protective effects in several RCTs [85–88].

### Rapid glycemic reduction

Rapid glycemic reduction should be avoided in people with chronically severe hyperglycemia. Investigators reported rapid changes in glycemic control led to early worsening of nephropathy, equivalent to retinopathy and neuropathy, which share similar mechanisms as microvascular complications [89]. Another study enrolled 2599 patients with T2DM also found a significant downward trend in eGFR change alongside an annual HbA1c reduction ≥3.0% [43]. These results suggest sustained monitoring and cautious interpretation of glycemic control, HbA1c, and eGFR changes are in need in clinical practices.

## Hemodynamic factors

### Hypertension

Hypertension significantly relates to early decline in renal function and is one of the main causes of chronic kidney disease [90,91]. Hypertension causes oxidative stress, inflammation, and subsequent glomerulosclerosis and atherosclerosis in the kidney, which contributed to renal function deterioration [92]. A longitudinal study in Japan reported that in populations with diabetes mellitus, high systolic blood pressure was associated with rapid decline in renal function [22]. Other studies in Italy and Taiwan also found hypertension to be a significant independent predictor for rapid decline of eGFR [38,44]. Nevertheless, the effect of blood pressure control on eGFR decline prevention is controversial. The Action to Control Cardiovascular Risk in Diabetes Blood Pressure (ACCORD-BP) trial, a randomized controlled trial, found that intensive blood pressure (BP) lowering (systolic BP < 120 mmHg), compared to a less intensive BP target (systolic BP < 140 mmHg), resulted in a more rapid decline in eGFR [45]. But subsequent longitudinal analysis found that intensive BP control related eGFR reductions did not associate with an increase in injury marker levels and assumed that eGFR decline observed with intensive BP goals in ACCORD participants may predominantly reflect hemodynamic alterations [93]. A *post-hoc* subgroup analysis including 1966 SPRINT (Systolic Blood Pressure Intervention Trial)-eligible ACCORD-BP participants and SPRINT participates with prediabetes suggests that intensive SBP lowering does not increase the risk of major adverse kidney events in individuals with T2DM [94].

### Intensive blood pressure control

A sharp decline in eGFR during BP lowering can be harmful to kidney outcomes. An observational study categorized the exposure of eGFR decrease as >15 *vs.* ≤15% between baseline and month 4, and the randomization to intensive *vs.* usual BP control. They found the decreases in eGFR >15% in both BP control group were associated with a higher risk of kidney outcomes, while in group of eGFR decrease <15%, intensive BP control associated with a lower risk of the kidney outcomes [95]. The results reminded us of the intensive monitor of eGFR during BP control to avoid hypoperfusion and adverse outcomes in kidneys.

### Glomerular hyperfiltration

An absolute, supraphysiologic elevation in GFR is observed early in the natural history in 10–67 and 6–73% of patients with type 1 and type 2 diabetes, respectively [5]. Pathogenesis of hyperfiltration in diabetes is complex, comprising numerous mechanisms and mediators, with a prominent role for hyperglycemia, distorted insulin levels, and proteinuria, especially in early diabetes and prediabetes [96–98]. A retrospective observational cohort study enrolled type 2 diabetes patients and found the hyperfiltration group exhibited a higher rate of eGFR decline compared with the normo-filtration group (−2.0 ± 0.9 *vs.* −1.1 ± 0.9 mL/min/1.73 m^2^/year; *p* < .001). Particularly, when hyperfiltration was combined with albuminuria, the risk of renal function decline was further compounded (hazard ratio 1.76–3.11, all *p* < .001) [46]. Previous studies also generated a similar conclusion that glomerular hyperfiltration is an independent risk factor of rapid renal decline either in T1DM or T2DM patients [21,47,48]. Studies also found that in DM patients with normoalbuminuric, hyperfiltration results in higher risk of rapid decreased GFR [47,49]. These findings suggested that glomerular hyperfiltration can lead to rapid eGFR decline dependent of the proteinuria onset. However, the present studies haven’t generated a consensus on the definition of hyperfiltration. In that case, some studies also reached negative conclusion that glomerular hyperfiltration was not associate with impaired renal function development because they applied 140 mL/min/1.73 m^2^ as threshold for hyperfiltration [44,99]. A more well-recognized definition of hyperfiltration is required in future studies and whether amelioration of hyperfiltration is renoprotective is worth investigating. Clinicians are expected to pay more attention to eGFR surveillance to prevent treatment delay due to the appeared normal renal function.

Intensive managements of glucose and blood pressure have long been recommended in diabetic patients with blockade in pathways as SGLT2, RAAS, and so on. Abundant evidences have declared the harm of hyperglycemia and hypertension, as well as hyper-perfusion, on renal function. Recently, more studies focused on the defects of hypoglycemia and hypotension which also contribute to renal function deterioration. In clinical practices, a more cautious monitor and management is recommended for DKD patients to avoid potential contribution to rapid renal function decline.

## Diabetes complication-related factors

### Proteinuria

Proteinuria is the result of podocyte injury caused by elevated blood glucose and hyperfiltration [100]. The proportion of patients with diabetes who develop microalbuminuria is substantial while relatively fewer patients develop macroalbuminuria [101]. Macroalbuminuria enhanced risk of rapid renal function deterioration as well as death [101,102]. On the contrary, populations with non-albuminuric CKD showed a slower rate of eGFR decline [103]. Studies from Joslin clinic reported that in patients with T1DM, the proportion of rapid decliners was 3, 11, and 35%, respectively when confounded with non-albuminuria, microalbuminuria, or macroalbuminuria. The data was 7, 15, and 51% in patients with T2DM [50]. Another prospective, observational cohort study enrolled 1682 patients with T2DM, and found patients with microalbuminuria or macroalbuminiuria had significantly faster age-adjusted annual eGFR declines compared to normoalbuminuric patients after 10-year follow-up [38].

### Diabetic retinopathy

Diabetic retinopathy (DR) is one of the most common microvascular complications of diabetes mellitus. DR and DKD often show parallelism in the process of occurrence and development as the high overlap in disease-causing risk factors, predictors, pathogenesis, and some medications [104]. The positive correlation between DR and DKD suggests the necessity of DKD screen in DR patients, and vice versa. According to the results of clinical studies, the presence of DR in DM patients often relates to anemia, proteinuria, and rapid decline in renal function [57,105]. A newly published Mendelian randomization study also provided genetic evidence for the noninvasive nature of DR in predicting DKD [106].

An earlier observational study evaluated the effect of DR concomitance on the rate of renal function decline in type 2 diabetic patients [51]. With no significant difference in blood pressure, metabolic control, and plasma lipid profile, the prospective cohort study found the rate of renal function decline was much greater in patients with DR than in those without (−6.5 ± 4.4 *vs.* −1.8 ± 4.8 mL/year; *p* < 0.0001). Patients with DR also manifested a significantly increased rate of total protein and albumin excretion during the 6-year follow-up. And they needed more antihypertensive drugs compared with those without DR. Another retrospective study assessed the effect of DR severity upon CKD progression in T2DM patients [52]. Baseline DR severity associated with faster renal function decline and greater albuminuria progression. Particularly, non-proliferative DR had 2.9 times and proliferative DR had 16.6 times higher risk for CKD progression. The results reinforced the necessity of DR evaluation at the first visit and subsequent close monitor of DKD patients. The mechanisms account for the worse outcome of patients with DR are obscure now while more advanced glomerular pathology was observed in patients with DR [107]. More high-quality studies and researches were in need to further elucidate the impact of DR on RPDKD and promote improved clinical monitoring and interventions.

### Cardiovascular autonomic neuropathy

cardiovascular autonomic neuropathy (CAN) is one specific diabetic neuropathy, among the most prevalent chronic complications of diabetes. The earliest stage of CAN may be completely asymptomatic. Then early signs as reduced heart rate variability appear. Later, people may present with resting fixed-rate tachycardia, changes in blood pressure regulation overnight, orthostatic hypotension, and sudden death [108]. CAN is an important risk factor for cardiovascular complications, particularly heart failure, and even sudden cardiac death [109].

Clinical studies have revealed that CAN was a strong independent predictor of early progressive renal decline in type 1 and type 2 diabetes [53,54,110–112]. A recent study analyzed the association between baseline CAN and subsequent glomerular filtration rate (GFR) decline with data of type 1 diabetes from the Preventing Early Renal Loss in Diabetes (PERL) study and type 2 diabetes from Action to Control Cardiovascular Risk in Diabetes (ACCORD) study [55]. The authors found that participants with CAN experienced more rapid GFR decline and were shadowed with greater risk of ≥40% GFR loss events during follow-up in both T1DM and T2DM. CAN may profoundly activate the sympathetic nervous system, which plays a critical role in the pathogenesis of renal dysfunction including activation of intrarenal RAAS, intraglomerular hypertension, and hyperfiltration [113,114].

### Atherosclerosis

Large observational studies, including prospective cohorts, have confirmed that carotid intima-media thickness and atherosclerotic plaques are associated with renal function decline and albuminuria progression [56,115,116]. Observational studies in patients with T2DM also found that carotid intima-media thickness (CIMT) and plaques predict renal function progression [117,118]. The underlying mechanisms include shared risk factors as hypertension, renal artery stenosis, and activated RAAS [119,120].

A prospective, multicenter cohort enrolled 967 patients with T2DM and preserved renal function with a median follow-up of 6 years and investigated the association between carotid atherosclerosis and renal function decline [41]. The investigators define rapid renal function decline as eGFR decline over 3.3% per year and they found that the presence of carotid plaque was significantly higher in rapid decliners than non-decliners (23.2 *vs.* 12.2%, *p* < 0.001). The multivariable logistic regression analysis also revealed carotid plaque presence as an independent predictor of rapid renal function decline after adjustment for established risk factors as age, sex, diabetes duration, HbA1c, baseline eGFR, UACR, CVD history, and hypertension (odds ratio, 2.33; 95% confidence interval, 1.48–3.68; *p* < 0.0001). Moreover, the inclusion of carotid plaque apparently improved the model performance for rapid decliner discrimination (AUC 0.772 *vs.* 0.744, *p* = 0.016).

Carotid plaque marks atherosclerosis presence, and the atherosclerosis of intrarenal arteries can be speculated which can cause renal ischemia and rapid GFR decline independent of albuminuria or proteinuria [121]. However, the negative results in some studies relating CIMT with rapid renal function decline in both healthy and diabetic populations indicate the need for more studies to further illustrate the impact of atherosclerosis in different stages on renal function deterioration.

Generally, DR, CAN, and atherosclerosis are all typical vascular complications of DM. Particularly, the microvascular lesions indicated by DR and/or CAN are very likely occur in kidney at the same time. Despite some conflicting results in the association of atherosclerosis and rapid renal function decline, the routine examinations of vascular lesions are highly recommended for DKD patients.

## Others

### Anemia

Anemia used to be a sign of incipient renal fibrosis and is widely detected in various forms of CKD, including diabetes [122,123]. Despite the extensive conception that anemia is the result of kidney impairment, accumulating evidences suggest that anemia could also cause rapid decline in renal function. Researchers have found that systemic administration of PHD inhibitors, drugs for treating anemia, can alleviate tubulointerstitial injury in streptozotocin-induced diabetes [124]. Data from the Reduction of End Points in NIDDM with the Angiotensin II Receptor Antagonist Losartan (RENAAL) study revealed that in patients with T2DM while blood pressure was controlled, hemoglobin (Hb) level is an independent risk factor that predict loss of kidney function and ESRD [125]. Another retrospective study in Japan found that in patients with T2DM and biopsy-proven diabetic nephropathy, lower Hb levels were associated with renal events (requirement for dialysis or 50% decline in estimated glomerular filtration rate from baseline) and all-cause mortality, especially in patients with severe interstitial fibrosis and tubular atrophy [126]. The longitudinal analysis of data from the National Database of Health Checkups in Japan further revealed the association of rapid decline in renal function and lower Hb levels [22]. Compared to participants with Hb levels ≥13 mg/dL, those with Hb levels ≥11 and <13 mg/dL, or Hb levels ≥9 and <11 mg/dL, or Hb levels < 9 mg/dL showed significantly higher risk of rapid decline in renal function. The retrospective study carried out in China, reported in a population of 2570 T2DM patients that anemia is associated with worse renal function and is an independent risk factor for rapid eGFR decline in type 2 diabetes [57]. In populations with preserved renal function and normoalbuminuria, lower hemoglobin level was still a risk factor for rapid decliners [58]. These findings suggest the symptom anemia in patients with DM is worthy of caution for potential accompanied renal insufficiency. Furthermore, the relationship between hemoglobin and renal function is expected to be differentiated from renal anemia to better address the contribution of anemia to renal function decline.

### Lifestyle

Lifestyle modification is recommended for subjects either with DM or trace proteinuria or impaired renal function. Recently, a growing number of studies try to clarify the exact impact of healthy lifestyles on DKD progression. In 2021, a study in Japan observed that among subjects including with diabetes mellitus, healthy lifestyles, i.e., noncurrent smoking, healthy eating habits and body mass index <25, markedly reduced the risk of developing trace proteinuria, positive proteinuria, and eGFR decline ≥20% within 2 years [59]. In 2023, a nationwide study in Japan also found that in 40–74 years old populations with T2DM, specific lifestyle risk factors were associated with a rapid eGFR decline and called for active lifestyle modifications [60]. Specifically, skipping breakfast was associated with rapid decline in eGFR regardless of baseline renal function. In subjects with baseline eGFR >30 mL/min/1.73 m^2^, regular smoking was a risk factor, while in subjects with baseline eGFR <30 mL/min/1.73 m^2^, non-refreshed sleep was identified. Moreover, many published studies agreed that smoking is an established risk factor for DKD development and progression [127–130]. All these risk factors reminded us that combined modifications and improvement in healthy lifestyle, particularly tobacco cessation, play vital roles in protecting renal function and retarding DKD progression.

## Conclusions

According to the different progression of proteinuria and decline of renal function, DKD can be classified into four subtypes, which are classical phenotype, albuminuria regression, a rapid decline in GFR, and non-proteinuric or non-albuminuric phenotype. Recently, the rapid GFR decliners in DKD patients have been a research field of growing interest with a strong relationship to subsequent risk of renal failure and cardiovascular outcomes. Studies have shown various genetic, clinical, biochemical, and histopathological parameters are associated with GFR trajectory prediction, including hereditary, age, gender, lifestyle, glomerular hyperfiltration, proteinuria, anemia, CAN, DR, atherosclerosis, early-onset T2DM and improper monitor of blood pressure and glucose. These results demonstrated the necessity and benefits of multifactorial monitor and interventions in DM patients to prevent DKD progression and subsequent comorbidities. There exists considerable heterogeneity clinically and pathologically in rapid decliners of DKD patients. Till now, not many studies focused on the rapid GFR decline phenotype of DKD, and specific risk factors remain unraveled. In the future, more studies are needed to incorporate each individual’s lifestyle, genetic, clinical, and pathological features to better characterize the rapid GFR decliners in DKD. With large-scale data, machine learning technique can be utilized to identify renal function trajectories more accurately. Further researches exploring the specific mechanisms in contributing rapid renal function decline might help find the precision diagnosis and medication approaches for the benefits of DKD patients.
